# Feasibility of Distributing Rapid Diagnostic Tests for Malaria in the Retail Sector: Evidence from an Implementation Study in Uganda

**DOI:** 10.1371/journal.pone.0048296

**Published:** 2012-11-12

**Authors:** Jessica Cohen, Günther Fink, Katrina Berg, Flavia Aber, Matthew Jordan, Kathleen Maloney, William Dickens

**Affiliations:** 1 Harvard School of Public Health, Harvard University, Boston, Massachusetts, United States of America; 2 Department of Economics, Northeastern University, Boston, Massachusetts, United States of America; 3 Economic Studies, Brookings Institution, Washington, D. C., United States of America; 4 Johns Hopkins School of Public Health, Johns Hopkins University, Baltimore, Maryland, United States of America; 5 Clinton Health Access Initiative, Kampala, Uganda; Johns Hopkins University, United States of America

## Abstract

**Background:**

Despite the benefits of malaria diagnosis, most presumed malaria episodes are never tested. A primary reason is the absence of diagnostic tests in retail establishments, where many patients seek care. Malaria rapid diagnostic tests (RDTs) in drug shops hold promise for guiding appropriate treatment. However, retail providers generally lack awareness of RDTs and training to administer them. Further, unsubsidized RDTs may be unaffordable to patients and unattractive to retailers. This paper reports results from an intervention study testing the feasibility of RDT distribution in Ugandan drug shops.

**Methods and Findings:**

92 drug shops in 58 villages were offered subsidized RDTs for sale after completing training. Data on RDT purchases, storage, administration and disposal were collected, and samples were sent for quality testing. Household surveys were conducted to capture treatment outcomes. Estimated daily RDT sales varied substantially across shops, from zero to 8.46 RDTs per days. Overall compliance with storage, treatment and disposal guidelines was excellent. All RDTs (100%) collected from shops passed quality testing. The median price charged for RDTs was 1000USH ($0.40), corresponding to a 100% markup, and the same price as blood slides in local health clinics. RDTs affected treatment decisions. RDT-positive patients were 23 percentage points more likely to buy Artemisinin Combination Therapies (ACTs) (p = .005) and 33.1 percentage points more likely to buy other antimalarials (p<.001) than RDT-negative patients, and were 5.6 percentage points more likely to buy ACTs (p = .05) and 31.4 percentage points more likely to buy other antimalarials (p<.001) than those not tested at all.

**Conclusions:**

Despite some heterogeneity, shops demonstrated a desire to stock RDTs and use them to guide treatment recommendations. Most shops stored, administered and disposed of RDTs properly and charged mark-ups similar to those charged on common medicines. Results from this study suggest that distributing RDTs through the retail sector is feasible and can reduce inappropriate treatment for suspected malaria.

## Introduction

The importance of scaling up parasitological confirmation of malaria for effective and efficient malaria control programs is widely recognized [1], [2]. Efforts to increase access to malaria diagnosis have been fueled by a growing awareness of high levels of inappropriate malaria treatment and of the negative consequences of treating non-malarial illnesses with antimalarials. Reyburn et al. [3] find that, among Tanzanian inpatients admitted to the hospital for severe malaria, the case fatality rate was higher for those who were actually malaria-negative than for those who were malaria-positive based on blood film microscopy. In general, there is substantial symptom overlap between malaria and other common illnesses caused by viral and bacterial infections [4], [5]. High rates of presumptive treatment with antimalarials can also result in a substantial waste of public and private resources – particularly in the presence of subsidies for expensive treatments such as ACT [6], [7], [8], [9], [10], [11], [12]. Presumptive treatment can also accelerate the emergence of parasite resistance [13], [14], [15]. Finally, the absence of widespread parasitological confirmation of malaria has been likened to “working with a blindfold” in that it is difficult to know where to target resources and how to track progress in malaria control programs [14], [16], [17], [18]. For all these reasons, the WHO now recommends parasitological confirmation of malaria whenever possible in its malaria treatment guidelines [1].

The development and refinement of rapid diagnostic tests for malaria (RDTs) have substantially increased the ability to make diagnostic confirmation available at all levels of the health system, including the formal and informal private sector and community-based care [16], [17], [18], [19], [20]. RDTs have been shown to be highly sensitive and specific and to outperform microscopy in field conditions [21], [22], [23], [24]. Universal access to malaria diagnosis, however, is unlikely to be achievable through scale up of RDTs in the formal public health system alone. This is because many patients suspecting malaria seek treatment outside of the public sector, where facilities are often distant, suffer frequent stock outs and have limited operating hours [25], [26], [27], [28].

There has been considerable discussion around whether and how increasing the availability of RDTs in the retail sector should be pursued in connection to (or perhaps instead of) a subsidy on ACTs, such as the one implemented through the pilot Affordable Medicines Facility for Malaria [6], [7], [29], [30]. In the context of ACT programs, scaling up RDTs offers the opportunity to significantly improve the targeting of public funds. While RDTs have been distributed in Cambodia's retail sector since 2002 [28], little is known regarding the quality of implementation, including how well the RDTs are being stored by shops, how successfully shops are performing them, and the extent to which the tests are being used to guide treatment behavior. If RDT negative results are commonly ignored and antimalarials are prescribed anyway, or if antibiotics are prescribed at very high rates to those testing negative, the cost-effectiveness and public health impact of RDTs could be compromised [31], [32], [33]. Beyond uncertainty over the operational feasibility of RDT scale up and the use of RDTs to guide treatment recommendations, concern has been expressed over whether retail sector providers would have an economic incentive to promote and sell RDTs to their clients, considering the substantial share of revenue that is generated from antimalarials [11], [34].

The most extensive evidence on the feasibility of RDTs in the retail sector outside this study comes from the experience of Cambodia, where RDTs (Paracheck, brand name “Malacheck”) have been socially-marketed and available in drug shops since 2002 [28]. Population Services International manages the training of shops and distribution of the RDTs, selling the tests for $0.05 to wholesalers with a recommended retail price of $0.24. While significant bottlenecks in supply chain procurement were encountered, there is evidence that RDTs have become increasingly available in shops since the roll-out of this program [28]. Unfortunately, there is no evidence on how well the RDTs are stored by shops, on how successfully shops administer them, or on the extent to which the tests are used to guide treatment behavior. No study has analyzed the feasibility of similar programs in the sub-Saharan African context.

To explore the feasibility of retail sector RDT distribution in Uganda subsidized RDTs were made available to licensed drug shops in six Ugandan districtss upon successful completion of an RDT training program. RDT training uptake, sales and final prices to patients were monitored. RDTs were sold to shops through a Ugandan wholesaler. he intervention was designed to replicate an unrestricted market at the retail level. Shops were free to choose whether to stock RDTs and sell them to clients, free to choose the price at which the RDTs were sold and free to make treatment recommendations as they wished. The primary objective of this study was to assess the overall feasibility of RDT distribution in the retail sector from a supply side perspective, i.e. to assess both the willingness of shops to distribute RDTs and the overall quality of the resulting test administration.

## Methods

### 1. Ethics Statement

Ethical approval for this study was given by the Harvard School of Public Health (IRB Protocol # P19371-105) and the Uganda National Council for Science and Technology (Protocol # HS805). Written consent from the female head of household was obtained at baseline for the household surveys. This included consent to be followed up with on a monthly basis and consent to speak with another caregiver in the household if the primary female caregiver was absent for an extended period of time. Verbal consent was obtained at each follow-up household survey. Written consent to participate in the study was obtained from drug shop owners at baseline unless the owner lived in a distant district in which case verbal consent was obtained by phone from the shop owner and written consent was obtained from the primary shop attendant.

### 2. Study Context and Population

Malaria is responsible for 30–50% of outpatient visits and 9–14% of inpatient deaths in Uganda, making it one of the most serious health problems faced by the population [35]. Malaria prevalence in children under 5 in Uganda ranges from 7.4% in Kampala to 80% in the mid-Northern region [27]. This study takes place in districts in the mid-Eastern and Northeastern regions, where prevalence among children is 40–55%. Ugandan Ministry of Health guidelines recommend parasitological confirmation of suspected malaria prior to treatment, but presumptive treatment is still the norm, especially in rural health clinics [36]. Ugandans commonly seek treatment for malaria in the retail sector [25], [26], [27], with outlets ranging from small, informal, unlicensed shops and vendors to licensed pharmacies (with the latter present mostly in urban areas).

The study was conducted in six districts in Eastern Uganda: Budaka, Bukedea, Kibuku, Kumi, Ngora and Pallisa. The area is mostly rural, with a total estimated population of 1.3 million in 2011. Malaria is endemic in the region, with transmission rates of more than 100 infective bites per person per year [27]. Peak malaria incidence in Uganda corresponds to the two rainy seasons, one between March and May and the other between September and December.

### 3. Procedures

#### Pretesting

Piloting took place between November and January 2010, and the main study period occurred between March 2011 (baseline) and April 2012 (endline). This paper focuses on the first six months after RDT training, covering the period from June to December 2011. Analysis is restricted to the first six months since additional interventions that could affect RDT distribution were introduced after this period and will be explored future work. Uganda is an AMFm pilot country, and the first subsidized ACTs arrived in Uganda in late April 2011.

#### Sample Selection

The sampling frame for the study included all villages with at least one drug shop licensed and registered with the Ministry of Health in the six study districts. Out of the 92 villages listed, 67 villages were randomly selected for the RDT program. A total of 108 registered shops in these 67 villages were invited to participate in the RDT training. In order to monitor treatment behavior at the household level, all households in the selected villages were listed and 30 households from each village randomly selected for household surveys.

#### Randomization

**Figure 1:**
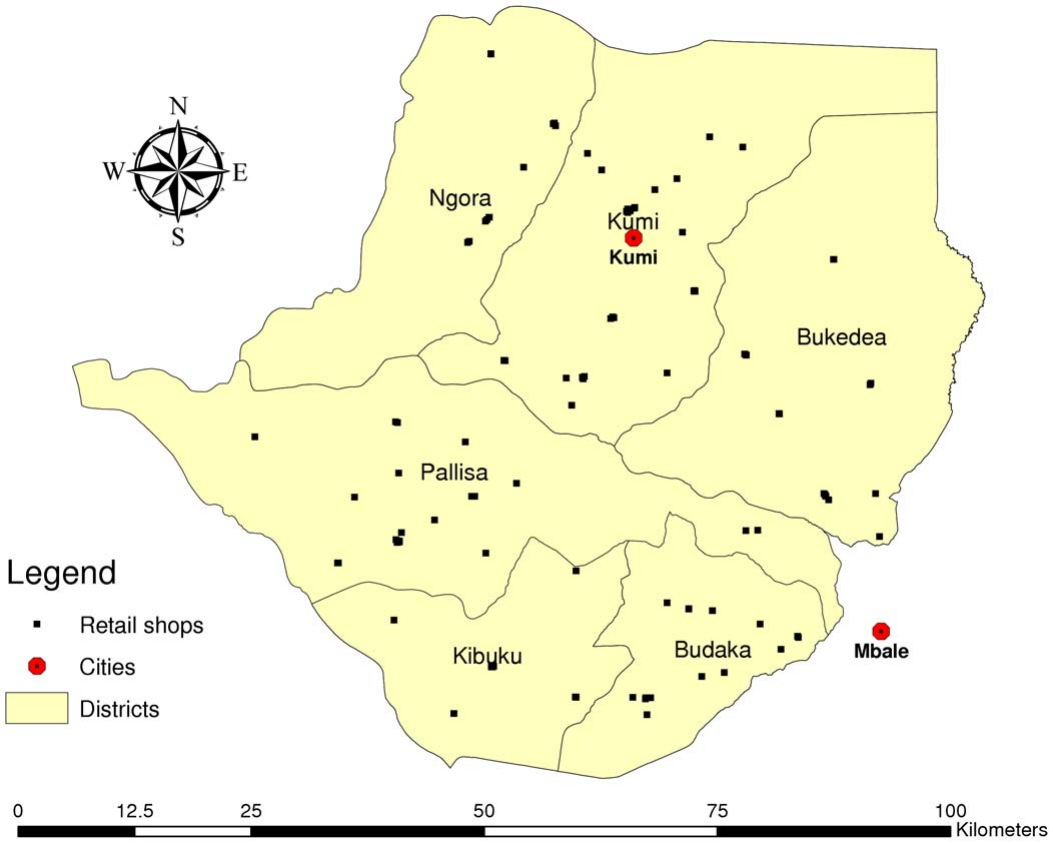
Study villages.

Both the selection of the 67 villages and the households within each of the villages were done using a simple random number draw generated by Stata Version 11.0 SE (Stata Corporation, College Station, TX). Figure 1 shows the spatial distribution of the selected villages within the six study districts.

#### Data collection

Drug shops were visited for an initial baseline survey and then once per month on an unannounced day by trained project staff for monitoring of RDT storage, administration and disposal. If none of the trained shop staff were present on the day of the visit, study staff made two additional attempts to complete the monitoring module in that month. At the time of the visit, information was recorded on how many RDTs (if any) the shop had purchased and administered in the past month, how many of the administered tests were positive and negative and what they were currently charging patients for an RDT. At the time of the monitoring visit, a free RDT was offered to a customer visiting the shop (or a volunteering passerby on the street if there were no customers in the shop) and a 17-point monitoring checklist was completed that covered whether shop attendant administered and read the RDT properly and disposed of sharps and other waste properly. The monitoring checklist also included items about how the RDTs were stored and the working surface on which RDTs were administered. Every three months (September 2011 and December 2011), four unused RDTs were collected from shops' current stock and sent for lot testing at the Foundation for Innovative Diagnostics (FIND) laboratory at the Pasteur Institute of Cambodia in order to ensure that the quality of the tests was not compromised. Lot testing involved measuring detection rates in parasite positive samples of 200 and 2000 parasites per micro-liter of blood, and measuring detection rates in parasite negative samples. As the RDTs distributed in the study arrived in Uganda in October 2010 (and were manufactured in September 2010) the tests had been in Uganda for 11 months at the time of the first round of lot testing and 14 months at the second round.

Households were visited for a baseline survey collecting data on demographic characteristics, assets, and malaria knowledge and treatment seeking behavior. After the initial visit, households were visited once per month for follow-up surveys, designed to capture illness episodes and how they were resolved. If respondents affirmed that someone in the household had been sick in the past month, an illness module was completed that collected detailed information on the type and duration of the health problem, type of health service accessed, medication taken, costs of medication and testing and whether blood-test-based diagnosis was conducted.

#### Equipment and Training

The RDTs used in this study were the *Care Start Malaria HRP2 Pf* test, manufactured by Access Bio. The most recent WHO/FIND report on RDT quality found that this test has a panel detection score of 98.7% and a total false positive rate of 2.4% [37]. We adapted the RDT training manual developed for training community health workers and medical professionals in rural health clinics in “generic Pf tests” by FIND, the World Health Organization (WHO)[38] based on initial field trials in Zambia [39] [40]. The manual was adapted to refer to the specific procedure for administering the CareStart test. The manual (and the training lessons) explained how RDTs work, how to administer them safely and how to read the results, and included pictorial instructions and exercises. It also reviewed signs of severe illness and when clients and children should be referred to a higher level health facility immediately. Participants were given only very broad recommendations on treatment and were not given any specific instructions or direction on when to perform the tests. The main recommendation was to “proceed as you would normally with a client you thought had [did not have] malaria” if the test was positive [negative]. No retail price was suggested. The objective was to train drug shop personnel only about how RDTs work and how to use them, and to observe whether and how shops chose to integrate them into their normal practice. The trainings were facilitated by trainers from the Ugandan Ministry of Health. The first day of training involved “classroom” instruction. The second day of training took place in a local health facility and provided the participants with practical experience administering RDTs to patients. Upon successful completion of the training, participants were given a free starter box of 40 RDTs and a sharps disposal box. Shops were also provided with free gloves for all RDTs purchased throughout the study.

#### RDT Distribution and Pricing/Markups

RDTs were purchased and imported for just under US$0.70 per test by the study. Upon arrival in Uganda, a sample of the procured RDTs was sent for lot testing at the Pasteur Institute of Cambodia. All sampled RDTs passed lot testing prior to the start of the study. The RDTs were sold to one of the main drug wholesalers (Abacus) in Kampala at a price of USH 300 (US$0.12) per RDT. A contractual agreement ensured that the RDTs would be distributed from Abacus's regional wholesale pharmacy in the city of Mbale, and sales would be restricted to study shops that had successfully completed the RDT training. To make sure this rule was enforced, sales staff had to enter the name of the purchaser as well as the quantity sold into a spreadsheet, which was collected by project staff on a bi-monthly basis. The agreed price at which shops were allowed to purchase RDTs from the wholesale pharmacy was USH 500 (US$0.20). In return for the USH 200 (US$0.08) margin per RDT, the wholesaler stored the RDTs in their main warehouse in Kampala, kept track of sales and restocked the regional pharmacy in Mbale as needed.

#### Additional Data Collection

In order to monitor shop behavior, the sales data reported by shops during the monthly monitoring visit was complemented with administrative records from the wholesale distributor in Mbale. Finally, a baseline survey was administered to drug shop owners (or, if the owner lived outside of the district, primary shop attendants), with basic questions about shop characteristics, questions about medication stocking, pricing and markups were asked, as well as questions about perceptions and beliefs about malaria treatment and diagnosis.

### 4. Data Entry and Analysis

Data was entered using the CSPro 4.0 package. All analysis was conducted using Stata Version 11.0 SE (Stata Corporation, College Station, TX).

## Results

### 1. Program Coverage

Using the official drug shop list provided by the District Assistant Drug Inspector, a total of 108 shops in 67 villages were identified in the study districts and invited to the training. 85% (n = 92) of invited shops attended and successfully completed the training; 15 shops had either closed or decided not to attend the training, and one shop failed the training. Out of the targeted 67 villages, RDTs were available through at least one shop in 58 villages (village level coverage 87%).

### 2. Characteristics of Trained Shops

Tables 1 and 2 show descriptive statistics for the 92 successfully trained shops as collected during the shop baseline interview. The average shop had been in operation for about 6.75 years and had only one employee in addition to the owner. Typically one person working at the shop was trained as a nurse or nursing assistant; only 12% of shops had a trained pharmacist actively working at the shop. As in most areas of Uganda, the density of drug shops is high, with enrolled drug shops on average reporting 5.6 other shops (most of which are likely unregistered/unlicensed) within a 30 minute walking distance. On average, drug shops report the nearest public health facility to be just over 30 minutes walking distance away. Most shops (87%) primarily stock from wholesalers in Mbale, which is the closest city. More nearby and smaller urban areas like Kumi are typically used for smaller purchases.

**Table 1:** Characteristics of Trained Shops.

Variable	Mean	(S.D.)
Months in operation	80.95	(59.25)
Number of employees (excluding owner)	0.99	(0.72)
Drug shops within 30 min walk	5.62	(3.61)
Minutes to walk to nearest public facility	34.28	(31.79)
Distance to Mbale in kilometers	42.48	(15.01)

**Table 2:** Characteristics of Trained Shops.

Variable	N	%
Most educated staff: nurse	36	39.13
Most educated staff: nurse assistant^)^	23	25.00
Most educated staff: pharmacist^)^	10	11.96
Most educated staff: other	21	23.91
Stocks from Mbalea)	79	86.96
Stocks from Kumia)	24	26.09
Stocks from other locationsa)	24	26.09

Notes:

a) Categories are not exclusive; some shops report to stock from multiple locations.

### 3. RDT Uptake and Volumes

Table 3 summarizes the total number of RDTs purchased by shops between July and December 2011. These are purchases made subsequent to the free box of 40 RDTs given to shops after successful completion of training. Over this six-month period, a total of 13,240 RDTs were sold, which corresponds to an average of about 2200 RDTs per month. As most shops are open 26 days per month on average, this corresponds to a rate of 85 RDTs per day, or about one RDT per day per shop. If the 40 RDTs given at training are included, this corresponds to 109 RDTs per day, or 1.2 RDTs per day per shop. Out of the 92 trained shops, 56 shops (61%) restocked at least once over the six-month period. As Table 3 shows, the variance between shops was large, with many shops selling small quantities of RDTs, and the largest six shops distributing over 40% of total volume.

**Table 3:** Restocking Distribution Over a Six Month Period.

RDTs Bought	Frequency	% of shops	Cumulative	Average RDTs per Day*
0	36	39.1%	39.1%	0.00
40	16	17.4%	56.5%	0.26
80	10	10.9%	67.4%	0.51
120	7	7.6%	75.0%	0.77
160	4	4.3%	79.3%	1.03
200	1	1.1%	80.4%	1.28
240	1	1.1%	81.5%	1.54
280	2	2.2%	83.7%	1.79
320	1	1.1%	84.8%	2.05
360	2	2.2%	87.0%	2.31
400	3	3.3%	90.2%	2.56
440	2	2.2%	92.4%	2.82
480	1	1.1%	93.5%	3.08
760	3	3.3%	96.7%	4.87
960	1	1.1%	97.8%	6.15
1160	1	1.1%	98.9%	7.44
1320	1	1.1%	100.0%	8.46

Note: RDTs were sold in boxes of 40, and therefore, total sales are in multiples of 40.

* Assumes shop is open 26 days per month.

**Figure 2:**
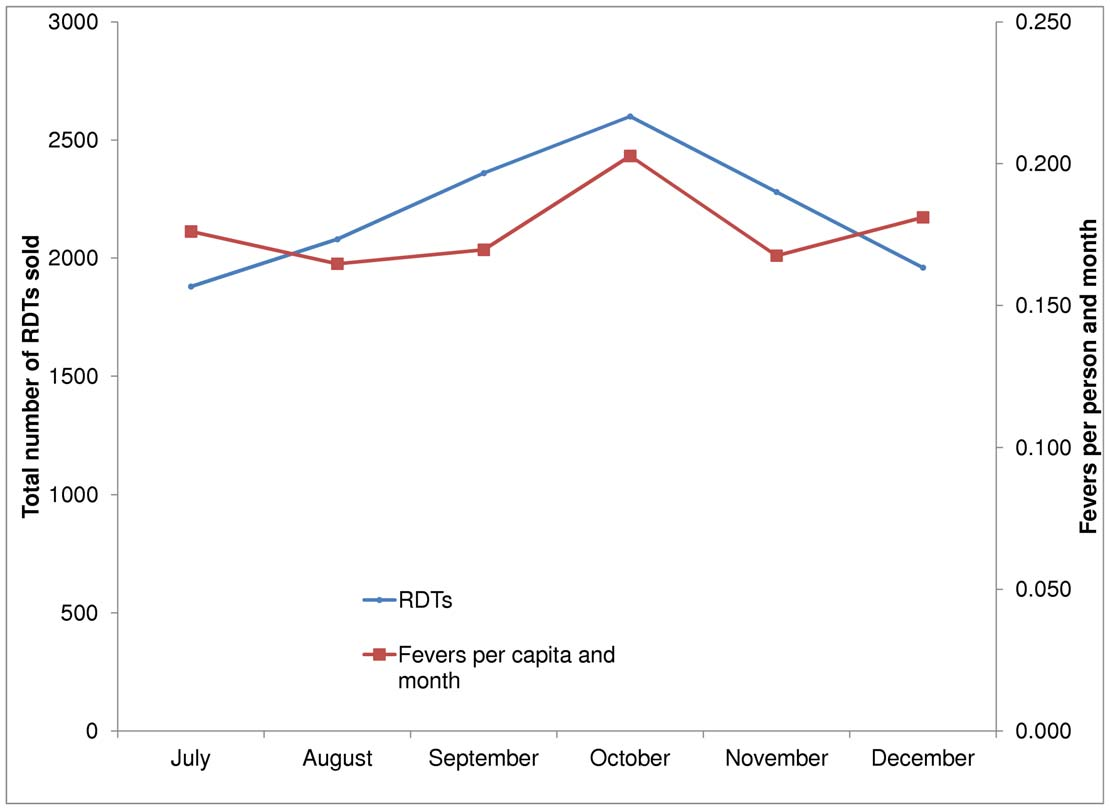
Trends in RDT Sales and Fevers.

Table 4 and Figure 2 show the number of RDTs purchased each month by trained shops from the wholesaler based on the wholesaler's administrative data. Restocking was highest in October, with a volume of 2600 RDTs, and lowest in July and December, with sales below 2000 tests. The incidence of fever over this period (based on the household survey data) fluctuated between 0.146 and 0.192 cases per person per month, with the highest levels in September and October, and lowest in August and November.

**Table 4:** RDT Sales by Month.

	Boxes sold	RDTs sold	Fraction of Shops Purchasing Any RDTs	Fevers per capita and month
July	47	1880	0.207	0.176
August	52	2080	0.261	0.165
September	59	2360	0.272	0.170
October	65	2600	0.315	0.203
November	57	2280	0.304	0.168
December	49	1960	0.239	0.181

### 4. RDT Pricing

Pricing information was collected both through the monthly shop monitoring visits and the household surveys. In both surveys, the median price was USH 1000 (US$ 0.40), which corresponds to a 100% markup for shops. It is possible that shops were charging this price to compete with local private clinics, which were charging USH 1000 for blood slide microscopy (see Table 5). Very few shops appear to have provided testing for free; the highest price households reported to pay was USH 5000 (US$ 2.00). Mark-ups for RDTs were in the mid-range of markups for other products at program shops. Based on information collected in our baseline survey with shop owners, participating shops charged on average a 140% markup on anti-pyretics, 108% markup on antibiotics, 65% markup on ACTs and a 79% markup on non-ACT antimalarials.

**Table 5:** RDT Prices.

	Household reports		Shop reports	
Price in USH	*N*	(%)	*N*	(%)
0	30	(6.30)	0	0.00
1–999	7	(1.47)	7	(2.99)
1000	263	(55.25)	184	(78.63)
1500	20	(4.20)	17	(7.26)
2000	89	(18.70)	20	(8.55)
2500	3	(0.63)	0	0.00
3000	23	(4.83)	1	(0.43)
>3000	10	(2.10)	0	0.00
Other/Don't know	31	(6.51)	5	(2.14)

Notes: Data reflects self-reports by households and shops over the monitoring period July to December 2011.

### 5. Program Compliance

Tables 6 and 7 illustrate that compliance with the treatment, storage and waste management protocols was very high overall. Compliance was particularly high with respect to storage and waste management: in 312 monitoring visits conducted between August and December 2011, overall compliance was very high, with only minor violations observed during the first rounds of the monitoring. As Table 6 illustrates, similarly positive results were found for the actual administration of the RDTs. On nearly all domains, compliance was over 95%. The only steps that resulted in non-compliance rates greater than 10% were: 1) the checking of the expiry date (most shops stated that they checked this at the time of purchase and thus did not check again at the time of administration), 2) the collection of the correct amount of blood with the pipette, and 3) the writing of the patient's name on the cassette. No single step in the administration process resulted in non-compliance rates greater than 17%. We also tracked compliance over time. As shown in Table 8, compliance improved slightly over time, with an average number of 0.79 specific tasks (out of the 17 tasks listed below) missed in August and an average number of 0.31 tasks missed in December.

**Table 6:** Compliance with RDT Storage and Waste Management.

Training Item	Compliance		Non-Compliance	
	N	(%)	N	(%)
Is the storage area dry and cool?	267	85.58%	45	14.42%
Is the working surface flat?	308	98.72%	4	1.28%
Does the DSV dispose of all used cassettes immediately?	277	88.78%	35	11.22%
Keeps the sharps container closed when not in use?	308	98.72%	4	1.28%
Area around the drug shop clean from used RDT products?	310	99.36%	2	0.64%

Notes: Data is based on 312 completed monitoring visits between July and December 2011.

**Table 7:** Compliance with RDT Administration Procedure.

	Compliance		Non-Compliance	
	N	(%)	N	(%)
All supplies available before performing RDT	274	99.28%	2	0.72%
Expiry date on test packet checked	229	82.97%	47	17.03%
Administrator wears gloves properly	272	98.55%	4	1.45%
Opens packet and removes contents	269	97.46%	7	2.54%
Writes the patient's name on the cassette	246	89.13%	30	10.87%
Cleans the patient's 4^th^ finger with alcohol swab	275	99.64%	1	0.36%
Allows cleaned finger to dry before pricking	276	100.00%	0	0.00%
Opens lancet and pricks patient's finger	276	100.00%	0	0.00%
Discards the lancet in sharps box after pricking	270	97.83%	6	2.17%
Use pipette to collect correct amount of blood	249	90.22%	27	9.78%
Uses pipette to place the drop of blood onto RDT	273	98.91%	3	1.09%
Discard pipette in the sharps box after placing blood on RDT	271	98.19%	5	1.81%
Places three drops of buffer into the round hole	275	99.64%	1	0.36%
Waits for 20 minutes before reading the results	266	96.38%	10	3.62%
Interprets the test results accurately	275	99.64%	1	0.36%
Records the results in registry	274	99.28%	2	0.72%
Disposes of cassette in sharps container	274	99.28%	2	0.72%

Notes: Based on 276 visits during which RDT administration was observed.

**Table 8:** Compliance with RDT Administration Procedure over Time.

	Average number of non-compliant behaviors	Fraction of shops with any non-compliant behavior
August	0.792	0.479
September	0.406	0.275
October	0.500	0.396
November	0.371	0.314
December	0.308	0.231

Notes: Based on 276 visits during which RDT administration was observed.

### 6. RDT Quality

Four tests from each of 61 shops (n = 244) and 55 shops (n = 220) were sent for quality testing in rounds 1 and 2, respectively. 100% of the RDTs passed lot testing (both parasite positive and parasite negative detection) in each round.

### 7. Adherence to Test Results

**Figure 3:**
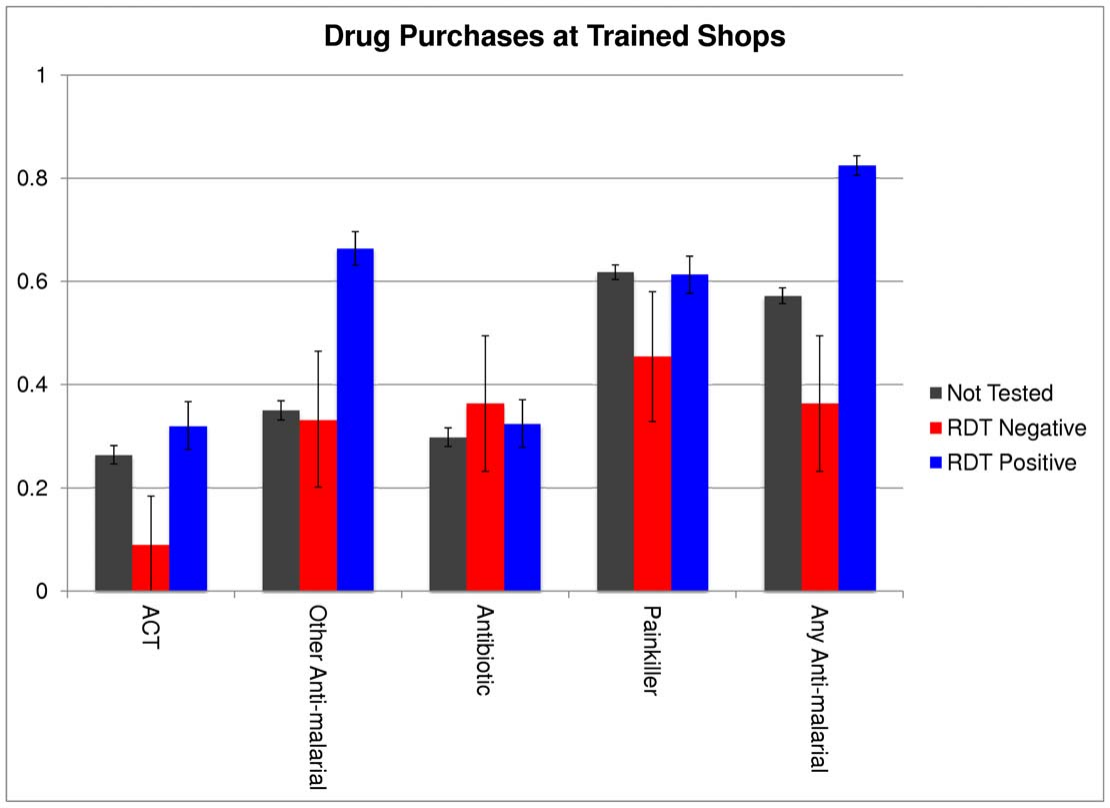
Percentage Purchasing Different Treatments by Diagnosis at Trained Shops.

Figure 3 shows medication purchases by diagnosis as reported by households visiting shops trained in the study between July and December 2011. Households reported a total of 2,037 visits and 307 of those visits involved the purchase of an RDT. Among RDTs purchased, 89% (247/307) reported a positive test result. Among those testing positive, 32% purchased an ACT, 23 percentage points higher than those testing negative (p = .005) and 5.6 percentage points higher than those not getting tested at all (p = .05). 66.4% of RDT-positive patients purchased another antimalarial of some kind, 33.1 percentage points higher than those testing negative (p<.001) and 31.4 percentage points higher than those not being tested at all (p<.001). There were no significant differences in rates of antibiotic purchases between RDT-positive patients and RDT-negative patients or between RDT-positive patients and patients who were not tested. Many patients bought several medications.

### 8. Program Cost

If the program evaluated here was scaled up exactly as is (including the extensive monitoring operations) to 500 shops, the estimated cost per RDT purchased and used would be US$1.59. This includes the subsidy to the manufacturer price, shipping and handling to get the RDTs to the distributor, the costs of training and supplying the drug shops with equipment needed to administer the tests (including gloves), the costs of monitoring the drug shops to be sure that they administer and handle the tests properly, and the administrative overhead for the project. Intermediate cost estimates assume that monitoring frequency is dropped to four visits per year, lot testing is done twice per year and can be done in a lab in Kampala (rather than shipping internationally) and that administrative duties can be spread over more shops. Under this scenario, the cost per test falls to US$1.02. The last cost estimate assumes that monitoring is done twice per year, that the price of RDTs charged by the manufacturer is 25% lower (reduced from US$0.69 to US$0.52 per test) and that shops contribute somewhat to the program cost by paying for their own food at training and by paying for the gloves that must be used with the RDTs. Under this scenario, the cost per test distributed through the program falls to US$0.59.

## Discussion

Overall, the results from this study suggest that it is feasible to distribute RDTs through the retail sector. All but one shop passed the training and compliance rates at monitoring visits were very high. Further, the majority of shops chose to continue stocking RDTs after the first free box ran out. Results from this study suggest, however, that there is significant heterogeneity in the desire to sell RDTs on the part of shops. Some shops enthusiastically adopted RDTs and promoted them to customers while others sold them only rarely or not at all. The reasons for heterogeneity are unclear – it is not known whether the differences are driven more by customer demand or shop owners' preferences. This is a question that deserves more study. The fact that some shops did not stock RDTs or sold them very infrequently suggests there may be a limit to the scale of coverage one can reach through a program such as the one explored here, at least in the short-term. One possibility is that coverage could have been increased if RDTs were available in smaller lots (the study required a minimum purchase of a box of 40). Future research will explore the impact of this intervention on community level coverage with RDTs and health outcomes.

Despite the variation in stocking behavior, RDT pricing was quite uniform, with 80% of shops reporting the same price of USH 1000. This price coincides with the price typically charged for microscopy in private clinics in the area, and corresponds to a 100% markup, similar to the markup these shops charge for commonly sold medicines. The household surveys confirmed that USH 1000 was the most common price for RDTs, though household responses displayed more variation, perhaps because of recall bias or difficulty distinguishing between the cost of the RDT and the cost of other components of treatment. This discrepancy could also arise if shops charge patients different prices for RDTs, but report to us only the price most commonly charged. Interestingly, this estimate of the market price for RDTs is very close to the estimated (stated) willingness to pay for RDTs (USH 1067) among drug shop customers in Uganda found in Hansen et al. [41].

In addition to providing encouraging evidence that shops can competently administer RDTs and have an incentive to sell them, results from this study provide suggestive evidence that RDTs will be used by shops and patients to guide treatment behavior in a beneficial way. ACTs and other antimalarials were significantly less likely to be purchased after a negative test result. This is encouraging evidence that RDTs could help reduce the cost of ACT subsidies or public support for malaria treatment more generally. The results also show that antibiotic purchases were higher for those testing negative than for those testing positive, though not significantly so. Considering the high rate of positive test results reported in the household survey—89% of patients taking an RDT at a trained drug shop reported a positive result—the results on treatment behavior conditional on test result should be interpreted with caution. The high rate of positivity among RDT takers may be interpreted as evidence that only patients with very serious symptoms of malaria get tested, but likely also represents a certain amount of recall bias among respondents. Two other possibilities for this high rate are that people may not have wanted to admit that they bought antimalarial medication without having a positive test, or that shop owners falsely told customers that they tested positive when they didn't in order to get them to buy antimalarials. When shop owners were asked about the frequency of positive tests their recall was that about 60% of tests were positive. The data collected did not allow to link patient's records to drug shop reports so that positivity rates reported by shops and households could not directly be compared.

Even though the results presented here suggest that rolling out RDTs in the retail sector is feasible, the overall reach of the program appears limited at this stage. The household data collected as part of this project suggest that 75% of fever patients sought treatment outside of their household. Among those who sought treatment, 53% did so at a drug shop (23% at a registered/licensed shop and 30% at an unregistered shop). Even from those fever patients who sought care at one of the trained shops, only 16% obtained a test. Overall, among fever patients sampled from our study districts who sought care in the retail sector, only about 6% got tested in one of the trained shops. Of course, if RDTs were sold to a higher fraction of customers at participating shops or if unlicensed shops were able to sell RDTs as well, coverage rates could increase substantially. Given that 45% of febrile illnesses in the sample were treated at retail shops, the fraction of total fever episodes that could be diagnosed through the retail sector is potentially large.

The results presented in this paper should be interpreted as short-term reactions to the introduction of RDTs on the part of shops and patients. RDTs were not found in any of the program shops at the time of the baseline interview and overall awareness of RDTs among both shop owners and patients was very low. This does not imply, however, that the study population was not familiar with malaria testing more generally. During initial focus groups, all participants indicated that they had heard of blood tests (slides), and a large majority of subjects reported that they had personally obtained blood tests at public health facilities. It is possible that, with more time, stocking rates of RDTs would have increased as shop owners became more comfortable with them and as patient demand increased. Of course, it is also possible that adoption was higher here than it would have been longer-term because shops were experimenting with a new product that they would later decide was not desirable or profitable. This project involved an information/education campaign that was rolled out after the study period explored in this paper. The role of this campaign on adoption will be examined in future work.

It could also be the case that particular aspects of the training program influenced adoption. Although the training program was presented in informational and value-neutral terms, it is possible that shop owners and other trainees considered the training to indicate that use of RDTs was socially desirable. As a result, those who participated in the study might have been more likely to sell them than a shop owner who was simply provided with the RDTs or one who had training from a company representative. On the other hand, if a national RDT training program were to be rolled out in shops, it is possible that more information on the social value of RDTs and the shop staff's responsibility to the community could boost adoption rates above what is seen here. It is also possible that our frequent monitoring of the drug shops increased their likelihood of selling the tests above what it would be if there was a lower level of supervision or no supervision at all.

Only licensed, registered drug shops were permitted to participate in this study. In many rural areas, the majority of shops available to consumers are not licensed. It is possible that the shops in this study were better able to implement the use of RDTs than the typical unlicensed shop would be. However, even if only licensed shops were able to successfully use and market RDTs that would greatly expand the availability of testing. There are several reasons why it might be desirable to limit RDT scale up programs to licensed shops, including the reduced monitoring burden from fewer shops and the benefits of containing the program to more “professional” establishments. On the other hand, allowing unlicensed shops to participate could expand the program's reach so the benefits and risks of expanding to this cadre of drug shops need to be weighed carefully.

While the study area was representative of rural and semi-urban areas in Africa, it is possible that the results would not extend to larger urban areas (where access to public facilities is higher). In addition, these results may not represent areas of lower malaria endemicity, as malaria treatment seeking is less frequent in places where malaria burden is low, but the value of the test for customers may be higher due to the reduced certainty about the nature of the illness.

Despite several potential limitations, this study demonstrates that a program distributing RDTs and providing training to drug shop owners is a practical way to encourage the adoption of this testing technology. The fact that unsubsidized RDTs were available for sale in licensed pharmacies in Mbale and other major cities, but that none of the program shops were selling them at baseline, suggests that the subsidy and training are necessary for promoting RDT sales in the retail sector. Overall this study demonstrates that there is a demand for RDTs among shops and patients and an ability among shop personnel to safely and effectively administer RDTs. This suggests that the retail sector is a viable channel for scaling up access to RDTs.
